# Supplementary material for: Astrocytic phagocytosis is a compensatory mechanism for microglial dysfunction
Source: EMBO J. 2020 Sep 22;39(22):e104464. doi: 10.15252/embj.2020104464 (PMC7667883; doi:10.15252/embj.2020104464)
Supplement: Supplementary file 1 — Appendix [file EMBJ-39-e104464-s001.pdf]

Astrocytic phagocytosis is a compensatory mechanism for microglial dysfunction

Konishi et al.

Table of contents

Appendix Figure S1. Quantitative morphometric analysis of microglia after microglial ablation.

Appendix Figure S2. Pieces of microglial debris were detected more clearly by staining for CD11b than Iba1.

Appendix Figure S3. Astrocytes contact microglial debris in *Siglech*$^{dtr/+}$ mice after microglial ablation.

Appendix Figure S4. MACS purification of hippocampal astrocytes for RNA-seq.

Appendix Figure S5. Very slight detection of microglia marker genes in RNA-seq data.

Appendix Figure S6. Attenuated expression of Iba1 in *Irf8*-deficient microglia.

Appendix Figure S7. No statistical change in the number of spontaneous apoptotic cells in mutant mice.

Appendix Table S1. Statistics of RNA-seq reads.

Appendix Figure S1

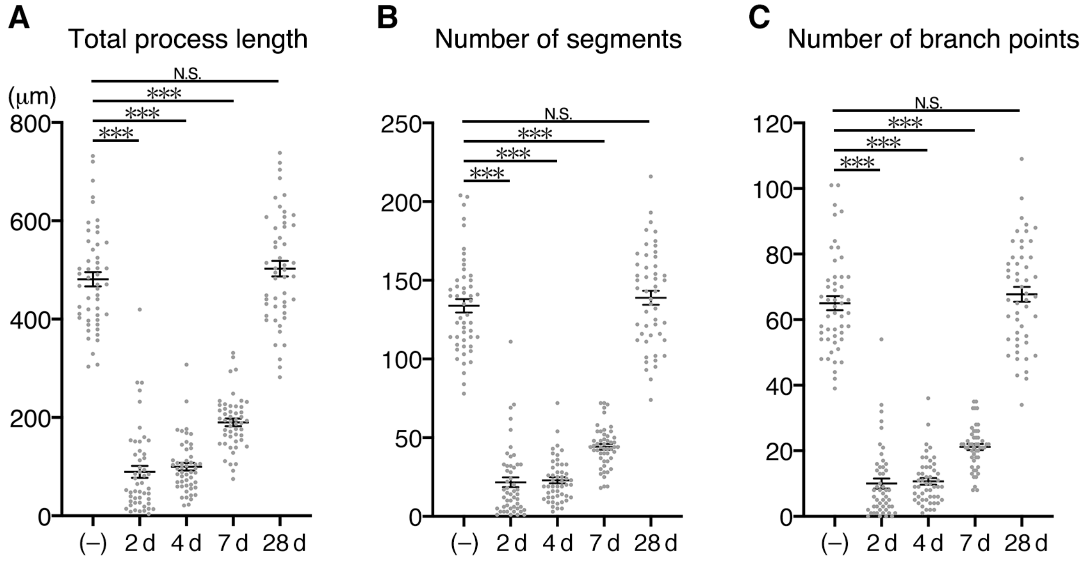

Appendix Figure S1. Quantitative morphometric analysis of microglia after microglial ablation.

A Total process length of a single Iba1$^+$ microglia in the hippocampal CA1 region of *Siglech*$^{dtr/dtr}$ mice after DT administration. (n = 50 cells from five animals per group).

B Number of segments of a single Iba1$^+$ microglia in the hippocampal CA1 region of *Siglech*$^{dtr/dtr}$ mice after DT administration. (n = 50 cells from five animals per group).

C Number of branch points of a single Iba1$^+$ microglia in the hippocampal CA1 region of *Siglech*$^{dtr/dtr}$ mice after DT administration. (n = 50 cells from five animals per group).

Data information: Values show the mean ± SEM. N.S.: no significance; ***$p < 0.01$; Mann-Whitney U test.

Appendix Figure S2

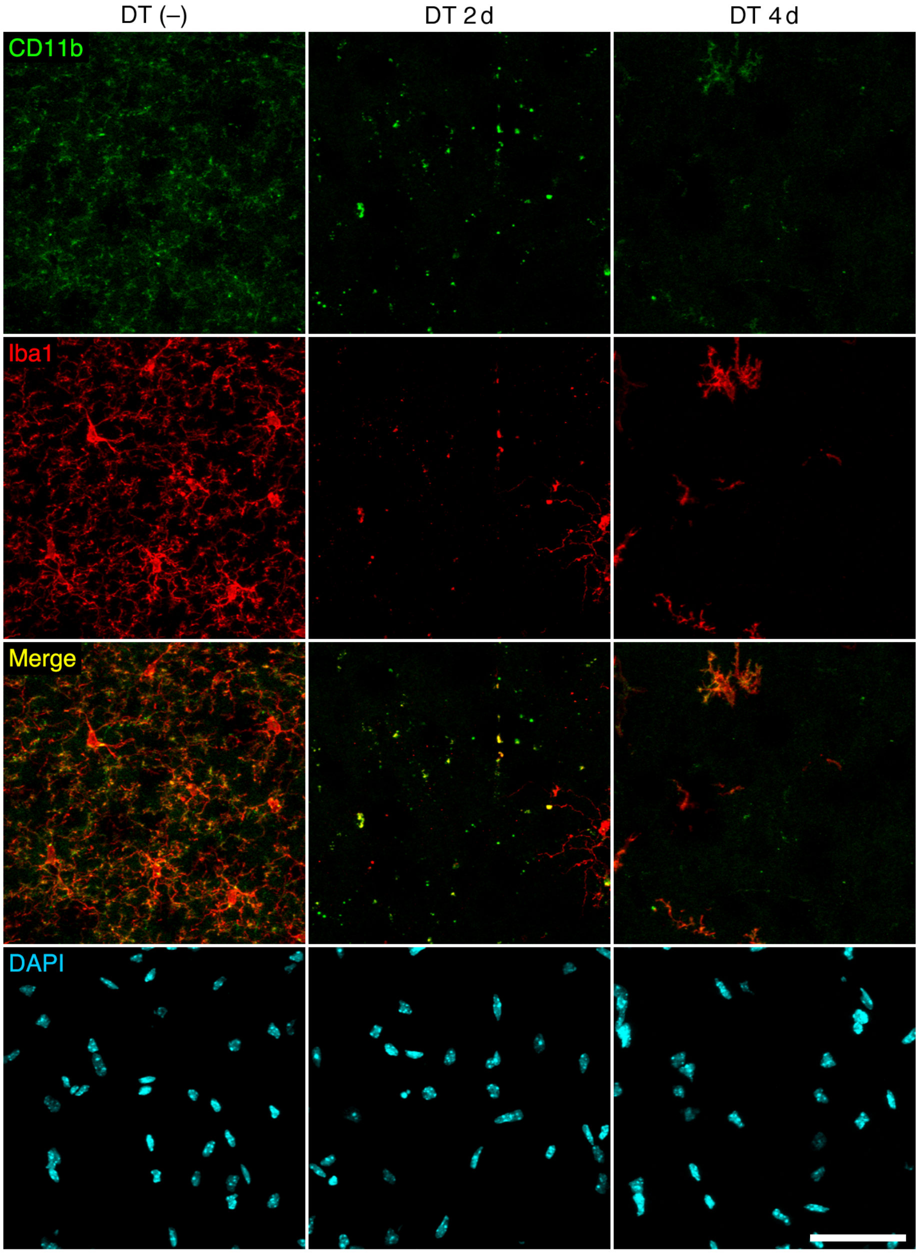

Appendix Figure S2. Pieces of microglial debris were detected more clearly by staining for CD11b than Iba1.

Sections were prepared from *Siglech*$^{dtr/dtr}$ mice at 0 (no injection), 2 and 4 days after DT administration, and stained with anti-CD11b (green) and anti-Iba1 (red) antibodies and DAPI (cyan). Representative images of the hippocampal CA1 region are shown. Scale bar, 50 µm.

Appendix Figure S3

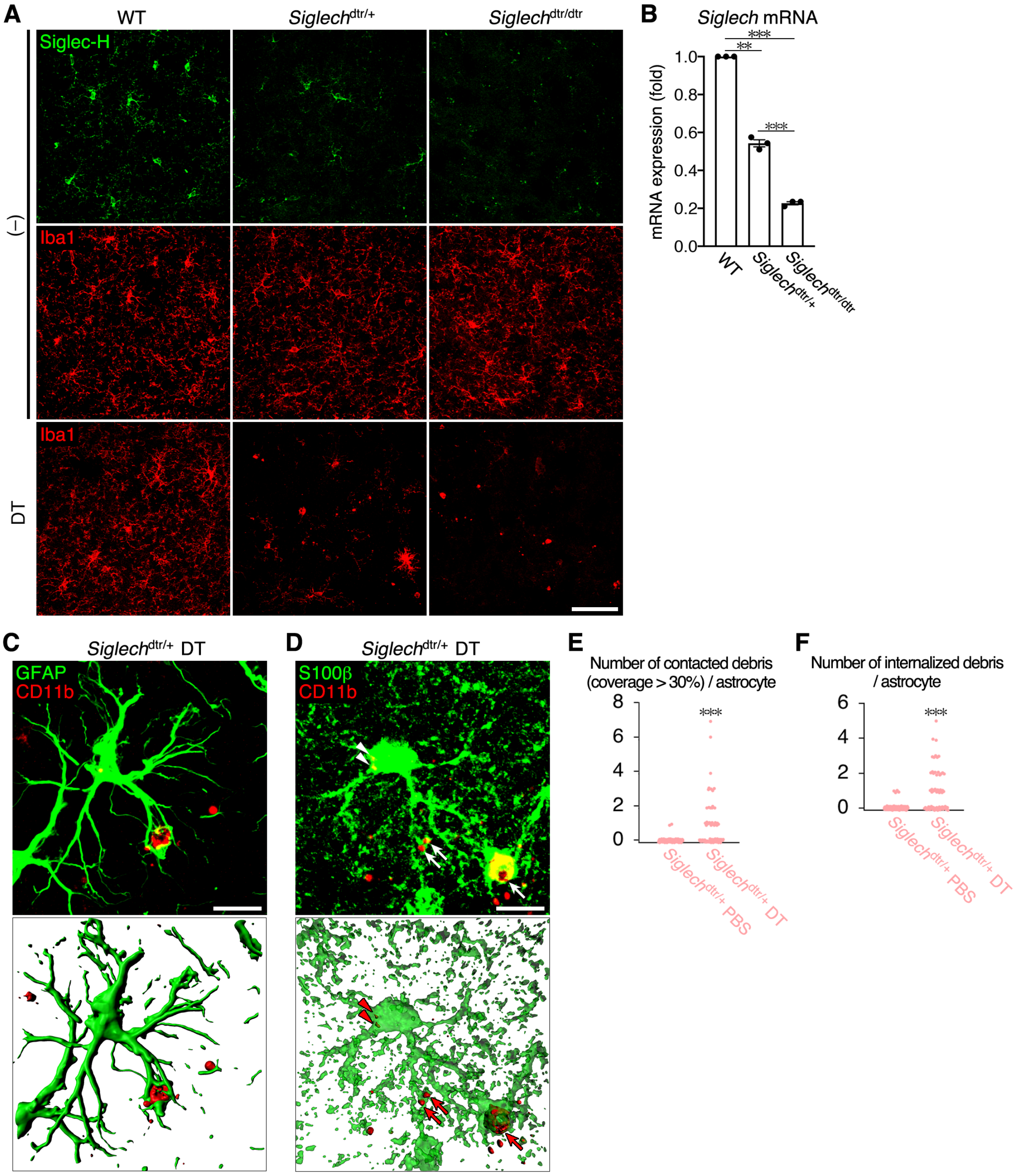

Appendix Figure S3. Astrocytes contact microglial debris in *Siglech*$^{dtr/+}$ mice after microglial ablation.

A Siglec-H expression and microglial distribution in hippocampal CA1 of WT, *Siglech*$^{dtr/+}$ and *Siglech*$^{dtr/dtr}$ mice. Sections prepared from non-injected or DT-injected mice (2 days after injection) were stained with anti-Siglec-H (green) and anti-Iba1 (red)

antibodies. Images were acquired using the same laser power and sensitivity, and image processing was the same for Siglec-H signals (green). Scale bar, 50 µm.

B Expression of *Siglech* mRNA in the hippocampus of WT, *Siglech*$^{dtr/+}$ and *Siglech*$^{dtr/dtr}$ mice analyzed by qPCR (n = 3 animals per group, one-way ANOVA with *post hoc* Tukey's test). Results are normalized to *Gapdh*, and are shown as ratios to the value of WT mice. Values show the mean ± SEM.

C, D Representative images of a phagocytic astrocyte in the hippocampal CA1 region of *Siglech*$^{dtr/+}$ mice 2 days after DT administration. Sections were stained with anti-GFAP (C, green) or anti-S100β (D, green) antibodies together with an anti-CD11b (red) antibody. 3D images (lower row) were reconstructed from confocal images (upper row) using Imaris software. Arrow: microglial debris contacted by astrocyte processes. Arrowhead: microglial debris internalized in astrocyte cytoplasm. Scale bar, 10 µm.

E, F Quantification of debris pieces (CD11b^{+} spheres with a diameter > 0.5 µm) contacted (coverage > 30%) (E) and internalized by (F) one astrocyte within 30 µm-thick sections (n = 75 cells from five animals per group, Mann-Whitney U test). The number of debris pieces was counted in 3D images reconstructed using Imaris software. All confocal images were acquired using the same laser power and sensitivity, and image processing was the same.

Data information: N.S.: no significance; $^{**}p < 0.01$; $^{***}p < 0.001$.

Appendix Figure S4

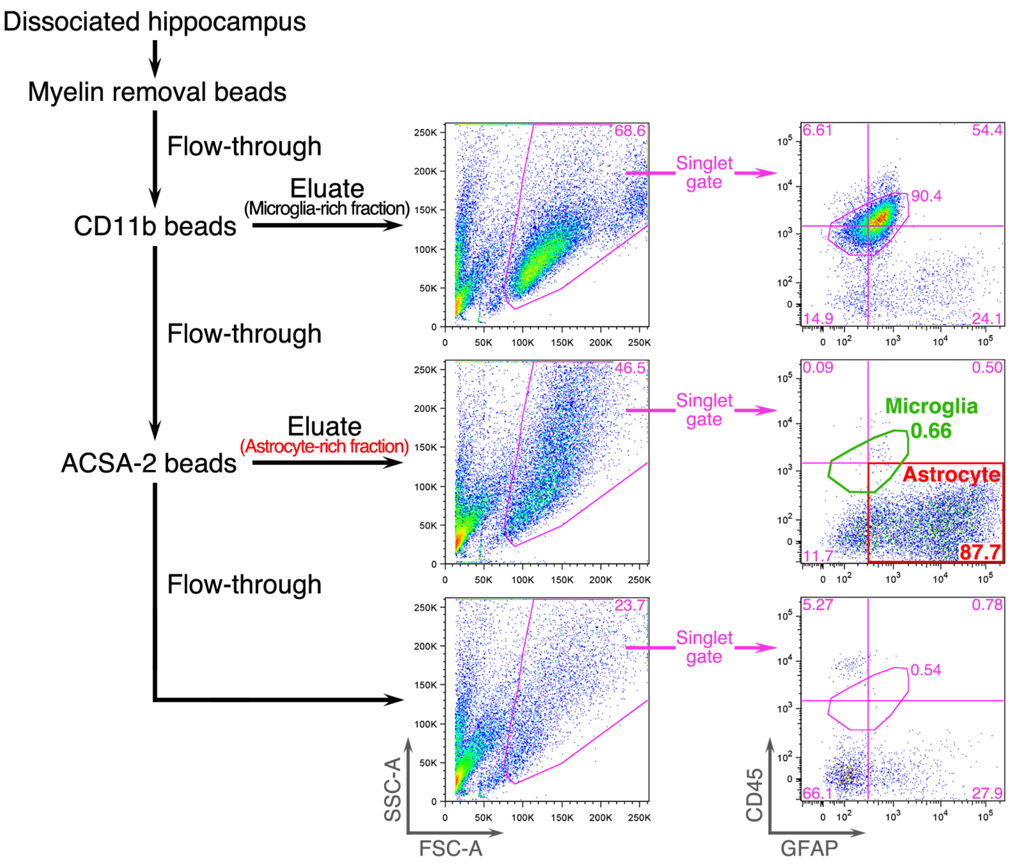

Appendix Figure S4. MACS purification of hippocampal astrocytes for RNA-seq. After removal of myelin debris using myelin removal beads, $CD11b^+$ microglia were depleted from the cell suspension to enhance the purity of astrocytes. Then ACSA-2^+ astrocytes were isolated from the $CD11b^-$ fraction. Cells in $CD11b^+$, ACSA-2^+ and the flow-through fractions were analyzed by flow cytometry to check purity. $CD45^+$ microglia were enriched in the $CD11b^+$ fraction. The ACSA-2^+ fraction contained abundant $GFAP^+$ astrocytes and very few $CD45^+$ microglia.

Appendix Figure S5

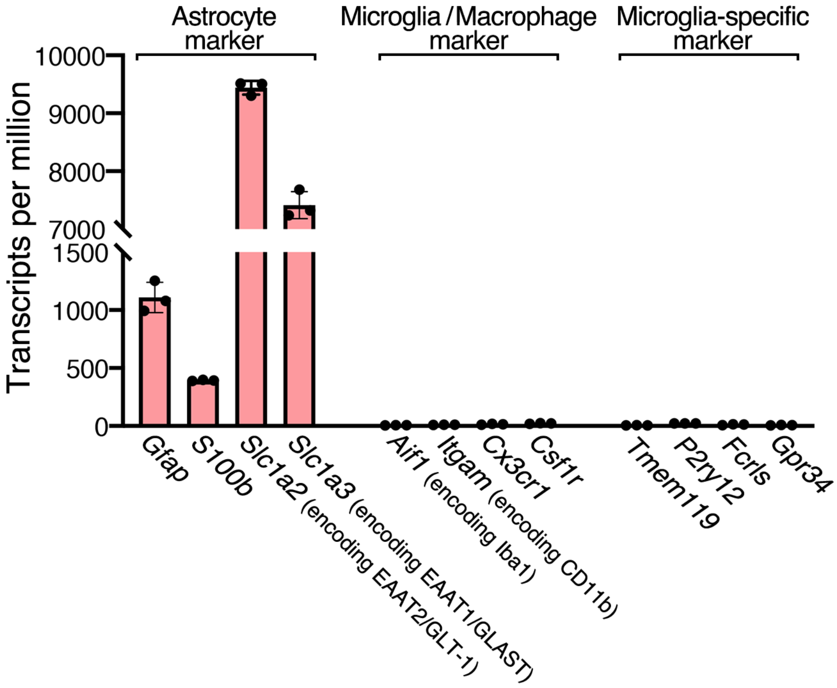

Appendix Figure S5. Very slight detection of microglia marker genes in RNA-seq data.

Expression levels of astrocyte, microglia/macrophage, microglia-specific marker genes in RNA-seq data. Values show the mean ± SEM.

Appendix Figure S6

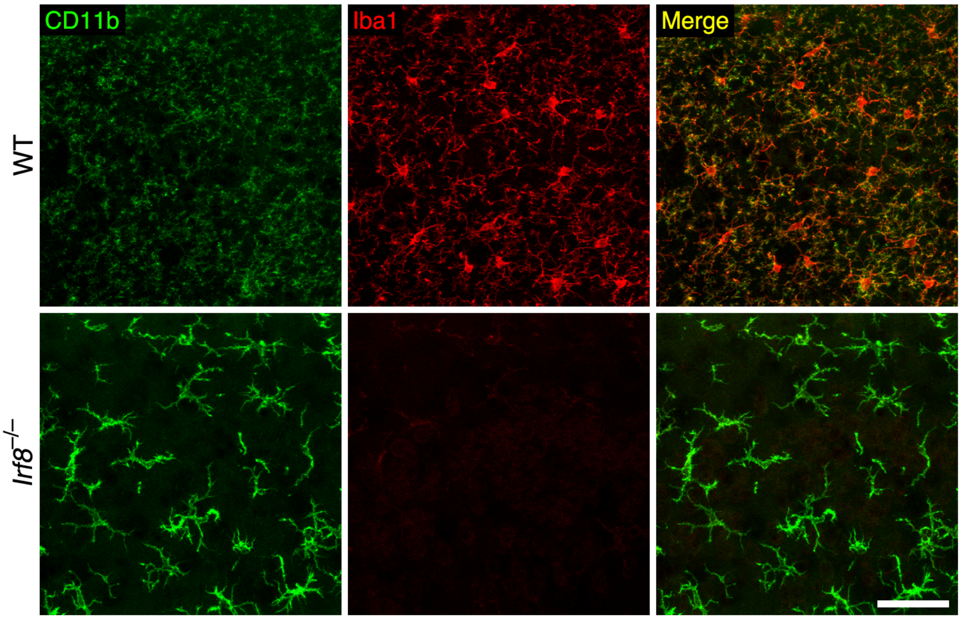

Appendix Figure S6. Attenuated expression of Iba1 in *Irf8*-deficient microglia. Sections prepared from WT and *Irf8*$^{-/-}$ mice at 3 w were stained with anti-CD11b (green) and Iba1 (red) antibodies. Representative images of cerebral cortex are shown. Images were acquired using the same laser power and sensitivity, and image processing was the same for CD11b (green) and Iba1 signals (red). Scale bar, 50 µm.

Appendix Figure S7

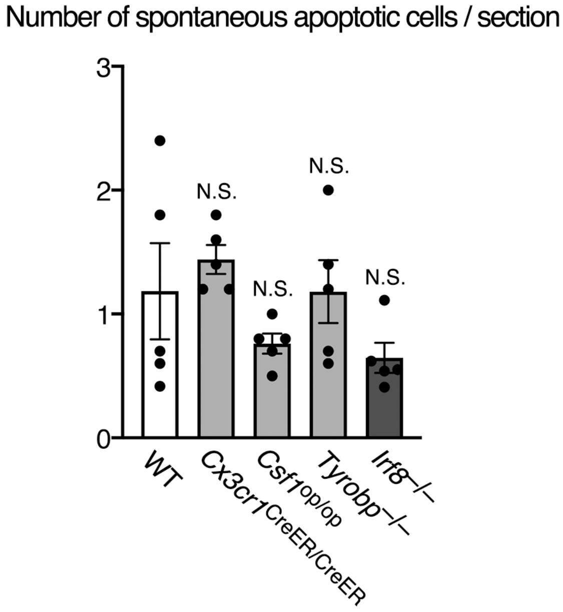

Appendix Figure S7. No statistical change in the number of spontaneous apoptotic cells in mutant mice.

The numbers of naturally apoptotic cells in bilateral cerebral cortex at 3 w were counted in the immunohistochemical images analyzed in Figure 7E and F (n = 5 animals per group, one-way ANOVA with *post hoc* Tukey's test). Values show the mean ± SEM. N.S.: no significance.

Appendix Table S1

Statistics of RNA-seq reads

	#Fragments	#Total reads	#Mapped	#Properly_paired
**PBS #1**	47,782,858	95,565,716	88,724,733	81,479,126
**PBS #2**	77,830,002	155,660,004	143,885,123	131,617,062
**PBS #3**	55,547,083	111,094,166	101,725,584	91,928,234
**DT #1**	50,025,661	100,051,322	92,617,244	84,791,324
**DT #2**	74,954,928	149,909,856	140,182,800	127,272,954
**DT #3**	53,740,608	107,481,216	100,248,046	90,496,514
