# Supplementary material for: Astrocytic phagocytosis is a compensatory mechanism for microglial dysfunction
Source: EMBO J. 2020 Sep 22;39(22):e104464. doi: 10.15252/embj.2020104464 (PMC7667883; doi:10.15252/embj.2020104464)
Supplement: Supplementary file 2 — Expanded View Figures PDF [file EMBJ-39-e104464-s002.pdf]

Expanded View Figures

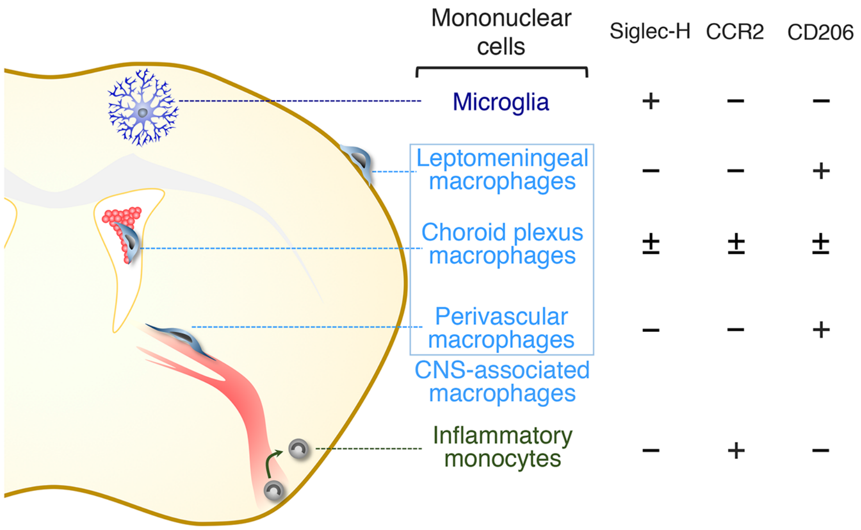

Figure EV1. CNS-related mononuclear cells and their marker molecules.

Siglec-H expression is almost confined to parenchyma-resident microglia. CCR2 is a near-specific marker for infiltrating monocytes. CD206 is specifically expressed by CNS-associated macrophages residing in the CNS boundary region (i.e., leptomeningeal macrophages, choroid plexus macrophages, and perivascular macrophages).

© 2020 The Authors

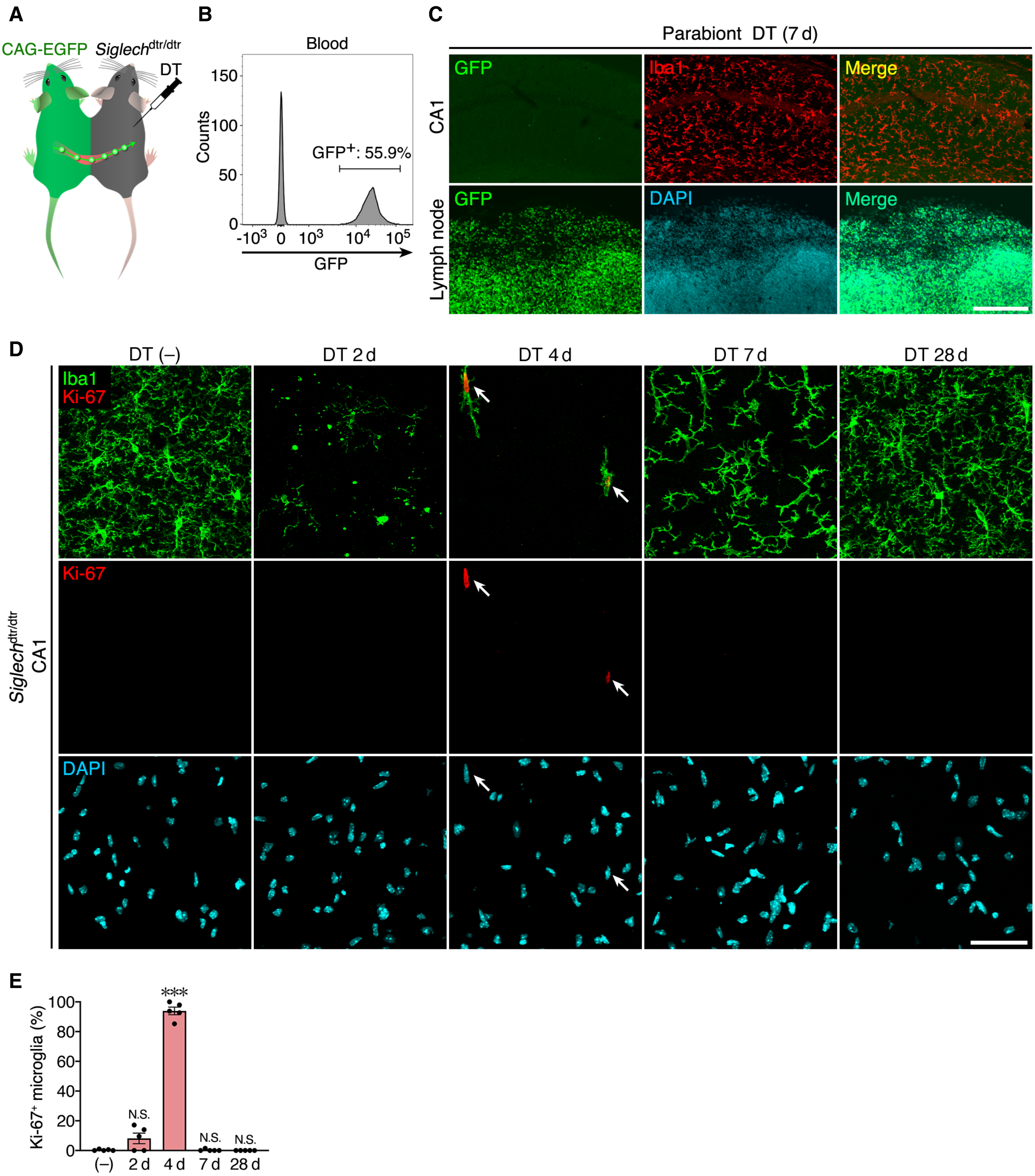

Figure EV2.

 © 2020 The Authors

Figure EV2. Survived microglia proliferate to replenish the brain.

A A schematic drawing of parabiotic coupling of *Siglech*$^{dtr/dtr}$ and CAG-EGFP mice.

B Flow cytometric analysis of blood chimerism of the parabiont. Representative data are shown.

C Images of hippocampal CA1 and lymph node of parabiont WT mice 7 days after DT administration. GFP signal (green), Iba1 immunoreactivity (red), and DAPI signal (cyan) are shown. Images were acquired using the same laser power and sensitivity, and image processing was the same for GFP signals (green). Scale bar: 200 µm.

D Time-course images of Ki-67 immunoreactivity in hippocampal CA1 of *Siglech*$^{dtr/dtr}$ mice after DT administration. Sections were stained with anti-Iba1 (green) and anti-Ki-67 (red) antibodies, and DAPI (cyan). Arrows indicate Ki-67^{+} proliferating microglia. Scale bar, 50 µm.

E Percentage of Ki-67^{+} microglia after DT administration (n = 5 animals per group, Kruskal–Wallis test with *post hoc* Dunn's test). Values show the mean ± SEM. N.S.: no significance; ***P < 0.001.

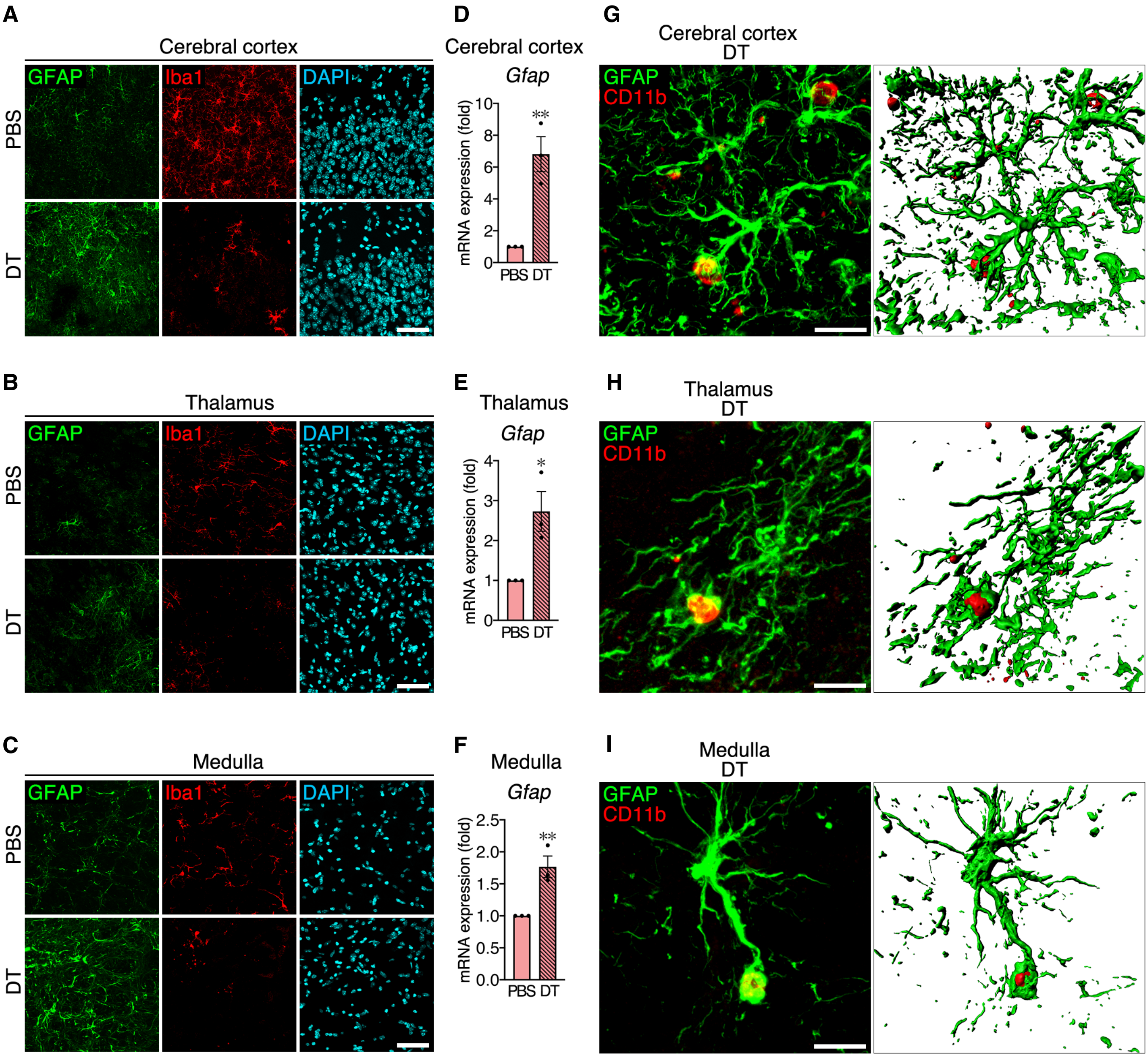

Figure EV3.

Figure EV3. Activated astrocytes contact microglial debris in other regions than hippocampal CA1 after microglial ablation.

A–C Astrocyte activation after microglial ablation in cerebral cortex (A; S1 area), thalamus (B; lateral posterior thalamic nucleus), and medulla (C; gigantocellular reticular nucleus). Sections were prepared from *Siglech*$^{dtr/dtr}$ mice 2 days after PBS or DT administration, and were stained with anti-GFAP (green) and anti-Iba1 (red) antibodies, and DAPI (cyan). Images were acquired using the same laser power and sensitivity, and image processing was the same for GFAP signals (green). Scale bar, 50 μm.

D–F Upregulation of *Gfap* mRNA in cerebral cortex (D), thalamus (E), and medulla (F) 2 days after DT administration to *Siglech*$^{dtr/dtr}$ mice (n = 3 animals per group, two-tailed unpaired Student's *t*-test). Results are normalized to *Gapdh* and are shown as ratios to the value of PBS-treated mice.

G–I Representative images of microglial debris surrounded by astrocyte processes in cerebral cortex (G; S1 area), thalamus (H; lateral posterior thalamic nucleus), and medulla (I; gigantocellular reticular nucleus). Sections were prepared from *Siglech*$^{dtr/dtr}$ mice 2 days after DT administration and were stained with anti-GFAP (green) and anti-CD11b (red) antibodies. 3D images (right panel) were reconstructed from confocal images (left panel) using Imaris software. Scale bar, 10 μm.

Data information: Values show the mean ± SEM. $*P < 0.05$; $**P < 0.01$.

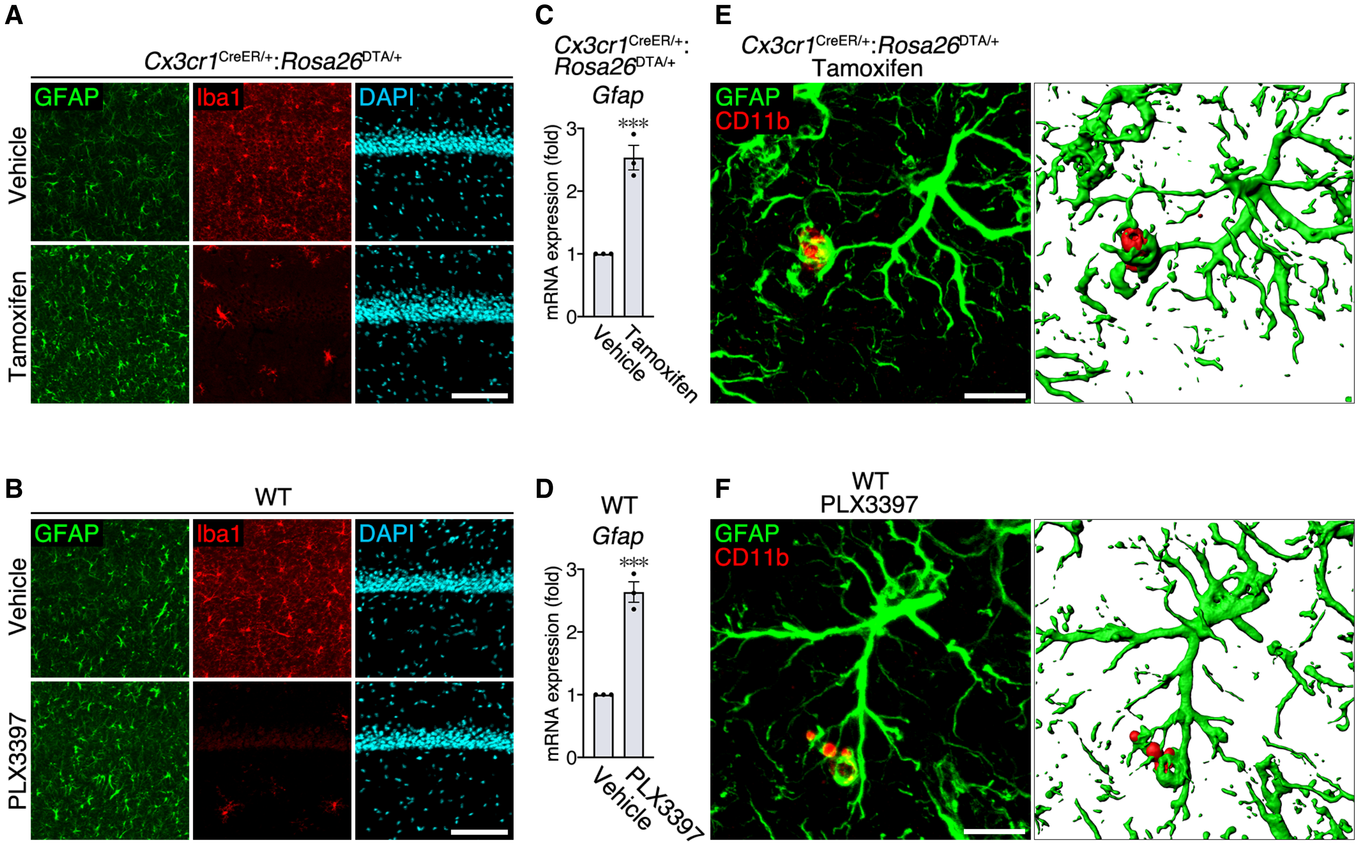

Figure EV4. Activated astrocytes contact microglial debris in other microglial ablation models.

A, B Astrocyte activation after microglial ablation in hippocampal CA1 after administration of tamoxifen to *Cx3cr1*$^{CreER/+}$:*Rosa26*$^{DTA/+}$ mice (A) or PLX3397 to WT mice (B). Sections were stained with anti-GFAP (green) and anti-Iba1 (red) antibodies, and DAPI (cyan). Images were acquired using the same laser power and sensitivity, and image processing was the same for GFAP signals (green). Scale bar, 50 μm.

C, D Upregulation of *Gfap* mRNA in the hippocampus after administration of tamoxifen to *Cx3cr1*$^{CreER/+}$:*Rosa26*$^{DTA/+}$ mice (C) or PLX3397 to WT mice (D) (n = 3 animals per group, two-tailed unpaired Student's *t*-test). Results are normalized to *Gapdh* and are shown as ratios to the value of vehicle-administrated mice.

E, F Representative images of microglial debris surrounded by astrocyte processes in hippocampal CA1. Sections were prepared from tamoxifen-administrated *Cx3cr1*$^{CreER/+}$:*Rosa26*$^{DTA/+}$ mice (E) or PLX3397-administrated WT mice (F), and were stained with anti-GFAP (green) and anti-CD11b (red) antibodies. 3D images (right panel) were reconstructed from confocal images (left panel) using Imaris software. Scale bar, 10 μm.

Data information: Values show the mean ± SEM. $***P < 0.001$.

 © 2020 The Authors

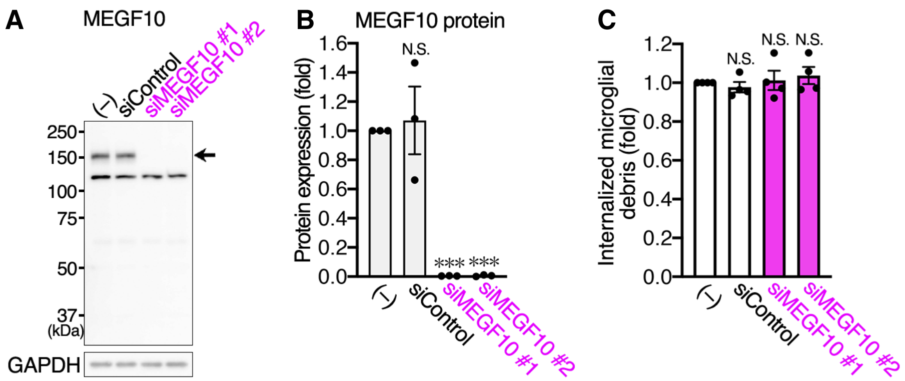

Figure EV5. MEGF10 deficiency does not affect uptake of microglial debris by primary cultured astrocytes.

A MEGF10 expression and its knockdown by siRNA in cultured astrocytes. MEGF10 expression in cultured astrocytes was examined by Western blotting. An arrow indicates the corresponding protein bands. GAPDH expression is shown as an internal control. MEGF10 expression was knocked down by two siRNAs.

B Quantification of siRNA knockdown in cultured astrocytes. Knockdown efficiency of MEGF10 was quantified from Western blots (n = 3 independent experiments, one-way ANOVA with repeated measures). Results are normalized to GAPDH and are shown as ratios to the value of non-transfected astrocytes.

C Flow cytometric analysis of uptake of microglial debris by cultured astrocytes. After MEGF10 knockdown, astrocytes were co-cultured with fluorescence-labeled apoptotic microglia, and fluorescence of internalized microglial debris in $GLAST^{+}$ astrocytes was quantified by flow cytometry (n = 4 independent experiments, one-way ANOVA with repeated measures). Results are shown as ratios to the value of non-transfected astrocytes.

Data information: Values show the mean ± SEM. N.S.: no significance; ***$P < 0.001$.

Source data are available online for this figure.

© 2020 The Authors
