# Supplementary figures and images for: Astrocytic phagocytosis is a compensatory mechanism for microglial dysfunction
Source: EMBO J. 2020 Sep 22;39(22):e104464. doi: 10.15252/embj.2020104464 (PMC7667883; doi:10.15252/embj.2020104464)

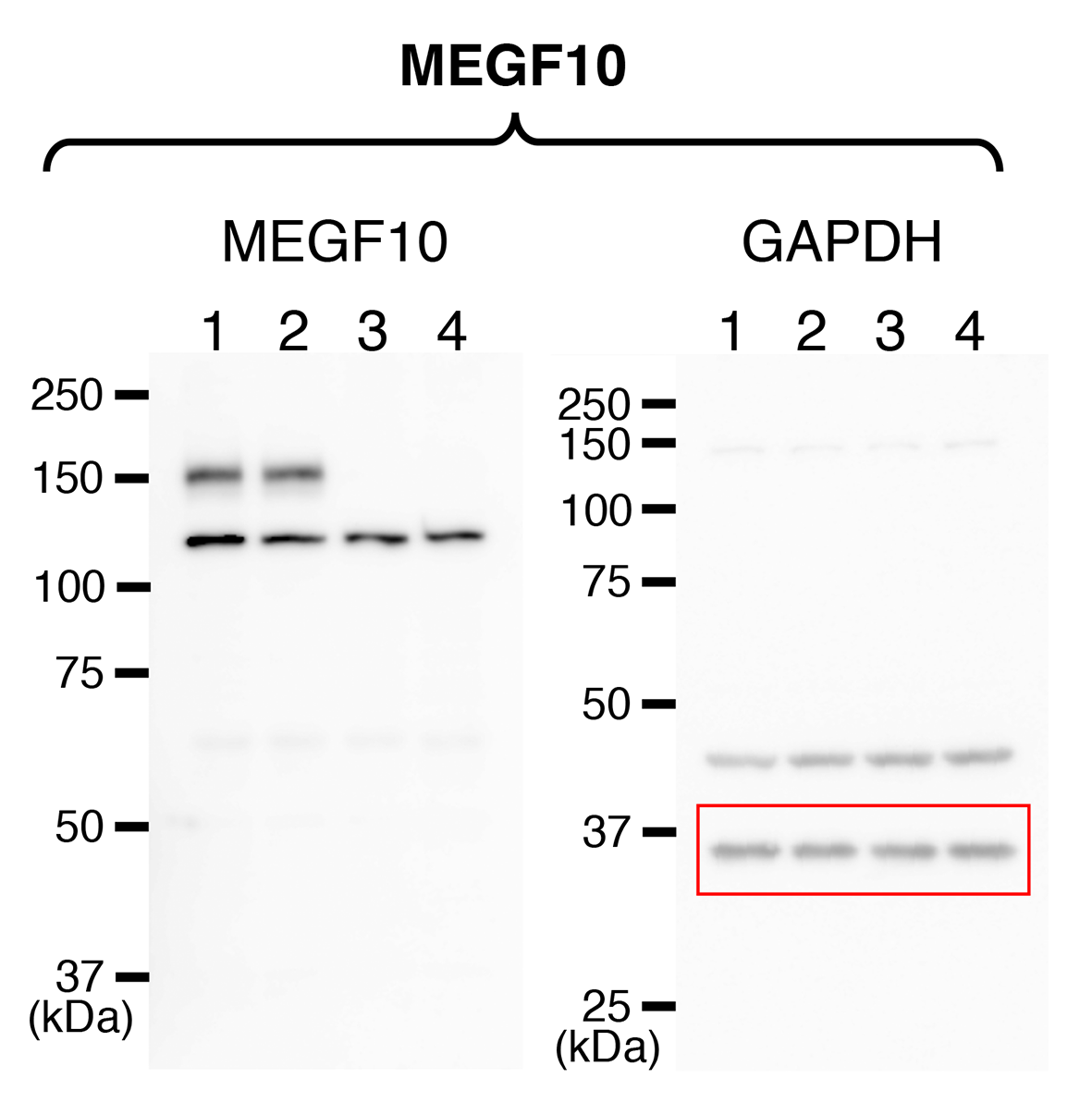

Supplement: Supplementary file 7 — Source Data for Expanded View [file EMBJ-39-e104464-s008.zip › embj2020104464-sup-0008-SDataFigEV/embj2020104464-sup-0008-SDataFigEV.tif]

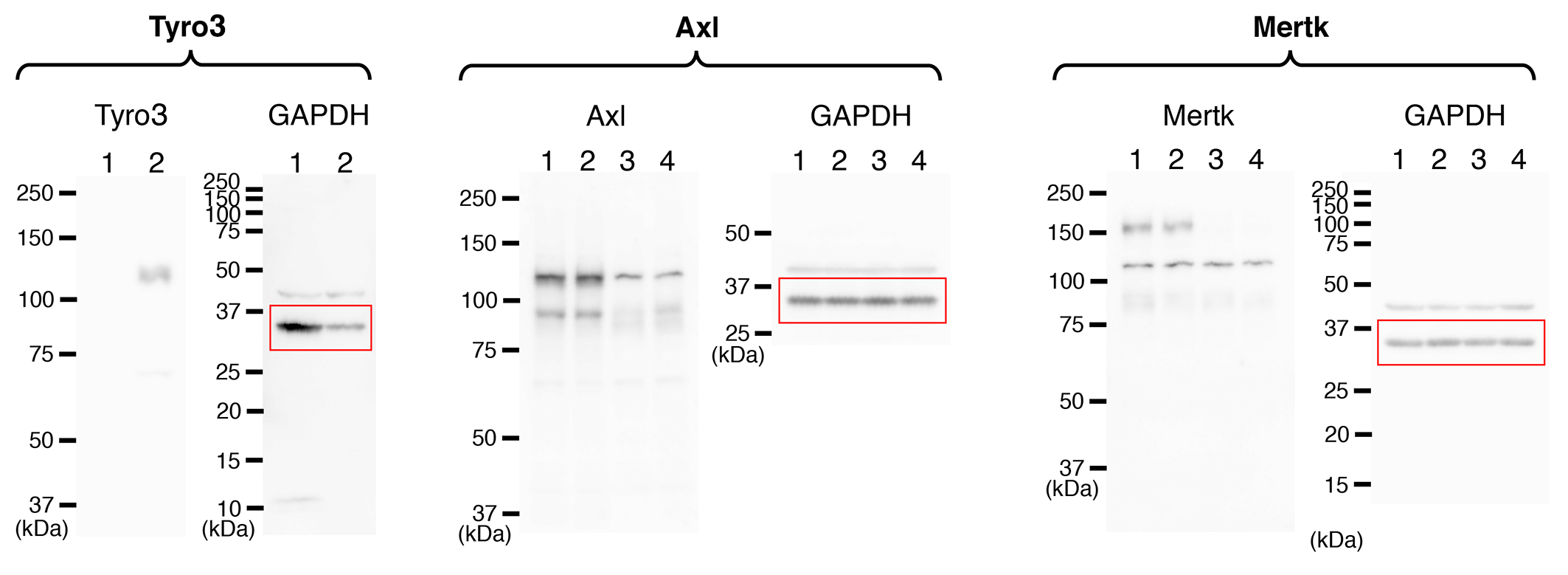

Supplement: Supplementary file 9 — Source Data for Figure 6 [file EMBJ-39-e104464-s007.tif]
